# A Putative New Role of Tv-PSP1 Recognizes IRE and ERE Hairpin Structures from *Trichomonas vaginalis*

**DOI:** 10.3390/pathogens12010079

**Published:** 2023-01-03

**Authors:** César Millán-Pacheco, Rodrigo Arreola, Alma Villalobos-Osnaya, Georgina Garza-Ramos, Iris N. Serratos, Adelaida Díaz-Vilchis, Enrique Rudiño-Piñera, María Elizbeth Alvarez-Sanchez

**Affiliations:** 1 Facultad de Farmacia, Universidad Autónoma del Estado de Morelos, Cuernavaca 62209, Mexico; 2 Psychiatric Genetics Department, Clinical Research Branch, National Institute of Psychiatry, Ramón de la Fuente, Calzada. México-Xochimilco 101, Colonia San Lorenzo Huipulco, Tlalpan, México City 14370, Mexico; 3 Posgrado en Ciencias Genómicas, Universidad Autónoma de la Ciudad de México (UACM), San Lorenzo # 290, Col. Del Valle, Ciudad de Mexico 03100, Mexico; 4 Departamento de Bioquímica, Facultad de Medicina, Universidad Nacional Autónoma de México, Universidad # 3000, Ciudad de Mexico 04510, Mexico; 5 Departamento de Química, Universidad Autónoma Metropolitana, Iztapalapa, Ciudad de Mexico 09340, Mexico; 6 Departamento de Medicina Molecular y Bioprocesos, Instituto de Biotecnología, Universidad Nacional Autónoma de México, Avenida Universidad 2001, Cuernavaca 62210, Mexico

**Keywords:** stem-loops, *Trichomonas vaginalis*, Tv-PSP1, REMSA, intrinsic fluorescence, molecular dynamics, molecular docking, binding free energy

## Abstract

To understand whether protein Tv-PSP1 from *Trichomonas vaginalis* recognizes mRNA parasite stem-loop structures, we conducted REMSA and intrinsic fluorescence assays. We found the recombinant Tv-PSP1 structure, determined with X-ray crystallography, showed unusual thermal stability of the quaternary structure, associated with a disulfide bridge CYS76-CYS104. To gain deeper insight into the Tv-PSP1 interaction with mRNA stem-loops (mRNAsl) and its relationship with thermal stability, we also used an integrated computational protocol that combined molecular dynamics simulations, docking assays, and binding energy calculations. Docking models allowed us to determine a putative contact surface interaction region between Tv-PSP1 and mRNAsl. We determined the contributions of these complexes to the binding free energy (ΔG_b_) in the electrostatic (ΔG_elec_) and nonelectrostatic (ΔG_non-elec_) components using the Adaptive Poisson–Boltzmann Solver (APBS) program. We are the first, to the best of our knowledge, to show the interaction between Tv-PSP1 and the stem-loop structures of mRNA.

## 1. Introduction

Gene expression regulation is critical in eukaryotic cells, which occurs at multiple levels by several complex mechanisms initiated inside the nucleus and completed in the cytoplasm. In these processes, proteins are capable of binding RNA to form a complex network with this molecule, recognized as sequences or stem-loop structures present in the 5′- or 3′-untranslated regions (UTRs) of target mRNAs [1]. 

Endonucleases [2] are proteins capable of recognizing single- or double-stranded RNA; their domain motifs have been characterized in other ribonucleases such as RNA III double-stranded RNA (dsRNA)-specific endonucleases [3]. Ribonuclease P is an essential endoribonuclease that processes the 5′ leader of pre-tRNAs; RNAs can interact near RNase P; and the cleavage depends on the nature of the interaction with the active site [4].

*Trichomonas vaginalis* is a flagellate protozoan that causes trichomoniasis, a sexually transmitted disease (STD) that is capable of infecting the urogenital tract of both men and women. The urogenital tract microenvironment affects the expression of key genes involved in parasite pathogenesis [5] in an iron-dependent manner. Iron regulates the virulence properties of some trichomonas probably via mechanisms mediated by iron-binding regulatory-like (cytoplasmic IRP-like) proteins interacting with hairpin-loop structures, which are called iron-responsive elements-like (IRE-like) structures found in the UTRs of target mRNAs such as *tvcp4*, *tvcp39*, and *tvcp12* cysteine proteinases [6,7,8]. *Tvcp39* contains an eIF-5A response-element-like stem-loop (ERE-like) structure in the 3′ UTR region [9]. *T. vaginalis* Tv-eIF5A, which is capable of binding eIF-5A response elements (EREs), binds specifically to the 3’ UTR of the *tvcp39* gene, suggesting its participation in mRNA expression and stability, where TvCP39 is involved in the cytotoxicity of *T. vaginalis* [10].

The L-PSP protein family possesses different biological functions [11,12,13] and possible activity as a ribonuclease [14,15,16]. A perchloric-acid-soluble protein (PSP) named Tv-PSP1 was previously reported in *T. vaginalis* [17]. We proposed that Tv-PSP1 has a putative ribonuclease function; however, additional evidence suggests that this activity is the result of a derivatization with chloride during the purification process, which is not a natural mechanism. The chlorination-induced ribonuclease activity of RidA (L-PSP) from *Staphylococcus aureus* has been reported [18,19] to maintaining the Tv-PSP1 RNAm binding function as a normal regulatory process associated with the protein complex. The L-PSP proteins from mammals inhibits cell-free protein synthesis by cleaving mRNA [14]. Notably, the ribonuclease activity might be an artifact of perchloric purification, or a kind of regulatory activity not well-described; in mammals, however, the participation of L-PSP proteins (L-PSP/UK114/HRSP12/RidA) in RNAm regulation mediates the interaction of a complex of proteins YTHDF2 (N6-methyladenosine reader protein), HRSP12 (L-PSP as an adaptor protein), and RNase P/MRP (endoribonucleases complex). HRSP12 recognizes RNA sites with the sequence GGUUC and weakly recognizes GCAAC; YTHDF2 binds to N6-methyladenosine; the HRSP12/YTHDF2/RNA assembly recruits the RNase P/MRP complex, leading to RNA cleavage [20].

Protein–RNA interactions are common in cellular processes in other organisms [2]; however, the L-PSP1 interaction mechanisms remain elusive. As such, our aim in this study was to validate and propose a putative interaction site between Tv-PSP1 and stem-loop structures via experimental and computational studies.

## 2. Materials and Methods

### 2.1. Transcription in Vitro

We obtained IRE-like and ERE-like sequences in vitro from *T. vaginalis* by PCR using primer sense 5′TAATACGACTCACTATAGGGCACATGTTCGTTCAGGCACCAT-3′ and antisense 5′CTTTCTGCTCATGTGCCTGAACGAACATGTG-3′; sense 5′TAATACGACTCACTA-TAGGGGTTTAGAATTTCCAATAA-3′ and antisense 5′ATTATGCTGAGTGAT-ATCCCCAAATCTTAAAGGTTATT-3′, respectively [6,9]. The sense primer contained a bacteriophage T7 promoter sequence and an additional GG sequence for enhancing transcription in accordance with a previously described method [9]. We used the PCR products as templates for RNA synthesis using an in vitro transcription MEGA short script kit (Ambion, Austin, TX, USA). We removed DNA templates and unincorporated nucleotides with DNase RQ1 (Promega, San Luis Obispo, CA, USA) treatment in the presence of RNase inhibitors (Promega, San Luis Obispo, CA, USA).

### 2.2. Purification of Recombinant TV-PSP1 and REMSA Assay

We purified recombinant Tv-PSP1 following a previously reported method, which we used in all assays [17]. We cultured *T. vaginalis* in yeast extract–maltose medium at pH 6.2 with 10% heat-inactivated horse serum (Gibco, Waltham, Brooklyn, NY, USA). We collected *T. vaginalis* by centrifugation at 300× *g* for 5 min at 4 °C, which we washed three times with PBS at pH 7.0. We resuspended the pellet in a buffer (50 mM TrisBase, 150 mM NaCl, 1 mM EDTA, and 1 mM DTT) with protease inhibitor cocktail (Roche, Basilea, Switzerland), which we then sonicated at a cycle of 80% amplitude for 30 s (on/off) and centrifuged at 10,000× *g* for 60 min at 4 °C. We added perchloric acid (Sigma-Aldrich, Santa Clara, CA, USA) to a final concentration of 5% (v/v) and then centrifuged at 10,000× *g* for 15 min at 4 °C. We precipitated the collected supernatant with 20% of ammonium sulfate (SigmaAldrich, Santa Clara, CA, USA). We resuspended the pellet in the same buffer described above. We spectrophotometrically quantified the concentrations of the proteins at A280 using a Nanodrop (Thermo Scientific, Waltham, MA, USA).

We used IRE-like and ERE-like sequences as templates for an REMSAassay. We incubated Tv-PSP1 (100 µg) and templates (1 µg) for 30 min at 37 °C in assay buffer (50 mM Tris-HCl, 75 mM KCl, 3 mM MgCl_2_, and 10 mM DTT). We performed an REMSA assay [9], we loaded the reactions in 1.7% agarose gel, which we had prestained with GelRed^®^ loading buffer. The electrophoresis running time was approximately 40 min at 90 volts. We obtained the images with a Chemidoc (Biorad, Hercules, CA, USA). We used an unrelated recombinant protein, GST, as a negative control. We conducted the experiments in triplicate.

### 2.3. Intrinsic Fluorescence Studies

We measured the fluorescence spectra from the protein solutions with a spectrofluorometer (ISS PC1, Champaign, IL, USA) equipped with a Peltier and water-jacketed cell holder for temperature control. We recorded the intrinsic fluorescence spectra of Tv-PSP1 (0.1 mg/mL) at an excitation wavelength set at 280 nm and bandwidths of 4 nm for both excitation and emission wavelengths. We also recorded the fluorescence intensity spectra as a function of incrementally adding aliquots of RNA ERE-like or IRE-like structures in 20 mM phosphate buffer and 90 mM NaCl. We incubated Tv-PSP1 with RNA, ERE-like, or IRE-like, at different concentrations (0.02–0.1µg/µL and 0.02-0.14µg/µL, respectively) for 30 min before we recorded the spectra; we subtracted the spectrum of the reference sample lacking protein. We obtained all spectra at 310 K, run for 2 min each. We conducted the experiments in duplicate.

### 2.4. Recombinant Tv-PSP1 Crystallization and Diffraction

We obtained recombinant Tv-PSP1 crystals using the sitting-drop vapor diffusion technique at 18 °C. We prepared the drops with a robot (Mosquito LCP, SPT Labtech) in 96-well IQ crystallization plates (SPT Labtech) with a mixture of 20 mg/mL Tv-PSP1 (0.3 µL) in 25 mM Tris-HCl, pH 7.4, 150 mM NaCl, and 1 mM DTT, with the crystallization solution (0.3 µL) containing 0.2 M ammonium sulfate and 0.1 M sodium acetate, at pH 4.6, and 30% PEG monomethyl ether 2000. Crystals suitable for diffraction appeared after 6 weeks and continued to grow for one month. We flash-cooled crystals by immersion in liquid nitrogen, exchanging the water with 10% PEG 400 into the mother liquor as a cryoprotectant. 

We collected diffraction data from one crystal (0.125 × 0.125 × 0.025 mm) at the ID19 beamline of the Advanced Photon Source, Argonne National Laboratory (Argonne, IL, USA) using a Pilatus 6 M detector. Reflections were indexed, integrated, and scaled with the XDS suite [21]. The crystal belonged to hexagonal space group P63 with cell dimensions a = 81.9 Å, b = 81.9 Å, c = 129.3 Å, and γ = 120° (Table 1). 

### 2.5. Tv-PSP1 Structure Determination by X-Ray

We determined the phases of the crystal structure via molecular replacement using the coordinates of perchloric-acid-soluble protein from *Rattus norvegicus* (PDB ID: 1QAH) [22] as the starting model, which we performed with Phaser [23]. We improved the phases by rigid body refinement and geometric constraint performed in REFMAC [24]. We refined the final model through alternating cycles of automatic and manual refinement with PHENIX [25] and COOT [26] to a final R of 19.5% (R_free_ of 24.1% calculated with randomly selected 5% of data) at 1.95 Ǻ (Table 1). The final crystallographic structure displayed good stereochemistry, as analyzed with MOLPROBITY [27]. We previously deposited the crystal structure with the Protein Data Bank with PDB ID 7KGC (Released on 20 October 2021).

### 2.6. Final Crystal Structure Analysis

We analyzed the macromolecular interfaces and assemblies of the asymmetric unit with PISA v. 2.1.0 (Qt-interface) software from CCP4 v. 7.0 [28]. We compared the monomers and trimers out with structure superpositions using UCSF Chimera software [29]. We used the Matchmaker tool in the structure comparison category for superpositions between monomers A/B, A/C, and B/C, with the best-aligning pair of chains between the reference and match structure, a cutoff of 0.6 A, and generating pairwise alignments. The superpositions between homotrimers TA/TB, TA/TC, and TB/TC were with specific chains in the reference structure with specific chains in the matching structure using a cutoff of 0.6 A. 

### 2.7. Molecular Dynamics Simulations of Tv-PSP1 Trimer Assembles

We assembled the final crystallographic structures of Tv-PSP1 as the biological units observed on the crystal lattice; we used trimers A, B, and C as, in the other members of the YjgF_YER057c_UK114 INTERPRO family, the biological unit is a trimer. Two structures (Tv-PSP1 trimer A and Tv-PSP1 trimer B with the presence of the disulfide bridge CYS76-CYS104) were almost identical but presented different average B factors (Table 2). We tested one structure, Tv-PSP1 trimer C (this structure does not have a disulfide bridge) in two possible situations: with no disulfide bonds present and with a double disulfide bond (Table 2). The additional double disulfide bond was considered in the CYS18-CYS101 residues because they are close to each other in loops from different subunits. The loops, as flexible structures, and both CYSs have a geometry that suggests a possible formation; however, we obtained no evidence of its formation. With this additional simulation, we explored a trimer with the most restrained conformations and a theoretical lower thermal stability.

We solvated all Tv-PSP1 trimer structures with a 10 Å cubic box water layer around each protein in 0.15 M NaCl using the CHARMM-GUI server (www.charmm-gui.org accessed on 23 October 2021) [30]. We obtained Gromacs configuration files from the CHARMM-GUI of each trimer for 100 ns at 30 °C and 1 atm. We simulated each system in triplicate on Gromacs 2019-2. Trajectory α-carbon root mean square deviation (RMSD), against the initial structure, showed some fluctuations in the first 60 ns (data not shown). We combined these 40 ns for each system into a single trajectory file (120 ns), and we obtained a single structure from clustering analysis. We implemented cluster analysis using the gromos method on the gmx cluster with a 1.25 Å radius on Gromacs 2019-2. 

We used two RNA IRE structures from human (ID: 1AQO [31] and 1NBR [32]) obtained by nuclear magnetic resonance. Both structures were energy-minimized via 100 steps of steepest descent with the CHARMM 40b2 program as well as by using the CHARMM36 force field parameterized for proteins [33].

### 2.8. Computational Docking: Interaction between Tv-PSP1 Trimer and Stem-Loop Structures

We conducted docking studies between Tv-PSP1 structures and mRNAs on the HDOCK server (http://hdock.phys.hust.edu.cn/ accessed on 6 July 2022) with default parameters [34,35,36]. The Tv-PSP1 trimer structures that we used in these studies were the cluster structures previously obtained. We removed all waters, ions, and ligands prior to protein–RNA molecular docking. As a result, the HDOCK server produced 10 protein–RNA complexes for each experiment. We energy-minimized each of these complexes (80 complexes) with 100 steepest-descent steps on CHARMM. We calculated the binding free energy of each complex as follows:

### 2.9. Binding Free Energy (ΔG_b_) of Tv-PSP1 Trimer–RNA: Electrostatic and Nonelectrostatic Contributions

#### 2.9.1. Electrostatic Calculations (ΔG_elec_)

We prepared docking structures to determine the electrostatic contribution in a continuous medium using the PDB2PQR program [37], in accordance with the Nathan Baker methodology. Electrostatic energy is divided in two components: main solvation and Coulombic:

(1) $$∆G_{\mathrm{elec}}=∆G_{\mathrm{solv}}+∆G_{\mathrm{Coul}}$$

where ΔG_solv_ is the solvation energies, and ΔG_Coul_ is the Coulombic energies of complex and free species (Tv-PSP1 and IRE). We incorporated the CHARMM force field [38,39] atom type and charges for all complexes. We obtained the electrostatics for each macromolecule by using the Adaptive Poisson-Boltzmann Solver program (APBS) solving the nonlinear Poisson–Boltzmann equation [38] with an ionic strength at 0.15 M (considering the ionic strength of the phosphate buffer used in the intrinsic fluorescence assays).

#### 2.9.2. Nonelectrostatic Calculations (ΔG_non-elec_)

We determined the Δ*G*_b_ contributed from the nonelectrostatic interactions as the energy released by hiding the interface area from the solvent when the complex formed: this energy is proportional to the change in solvent-accessible surface area (∆ASA) and a parameter such as the surface tension (γ), having a value of 0.021 kJ/mol*Ǻ^2^ for water: Δ*G*_non-elec_ = γΔASA_interface_ [40,41]. We required PDB files to determine ΔASA using Visual Molecular Dynamics (VMD) software:

ΔG_non-elec_ = γ (ASA_Tv-PSP1-IRE_ − ASA_Tv-PSP1_ − ASA_IRE_) (2)

Finally, to determine the ΔG_b_ for Tv-PSP1 trimer–IRE, we followed the same protocol as previously reported [42,43]:

ΔG_b_ = ΔG_solv_ + ΔG_coul_ + ΔG_non-elec_ (3)

## 3. Results

### 3.1. Crystal Structure and Thermal Stability

In the asymmetric unit, the final crystal structure (PDB ID: 7KGC) of Tv-PSP1 contains 4 monomers (P63 space group array, Table 1), 295 water molecules, and 3 electronic densities, so was identified as a TRIS buffer molecule. The asymmetric unit does not correspond to the biological unit; the latter is a homotrimer. The monomer D is not visible in a large part of the structure: only the fragments in contact with monomer A are visible. The reconstruction of the complete unit cell shows the crystal packing, allowing the formation of four different trimers. Each monomer of the asymmetric unit is part of one trimer formed by the same monomers from different asymmetric units (Figure S1).

Additionally, related to these monomeric distributions in the lattice, we observed unusual thermal stability of the quaternary structures (on each homotrimer), associated with the formation of the disulfide bridge CYS76-CYS104 (Table 3). The monomer contains another five CYS residues (18, 23, 74, 101, and 103), but they do not form an additional disulfide bridge. With respect to monomer folding, Tv-PSP1 maintains an antiparallel/parallel mixed beta sheet in order 123645, with two alpha helixes on one side, and the trimers form with closely packed beta sheets. In the structure descriptions in this paper, the “top” surface of the trimers is defined as the surface of the three-fold axis of the molecule, where the convergence of the three loops 107-115 (a trimerization structure) is located, and “down” surface corresponds to the opposite surface of the three-fold axis of the molecule, where the convergence of the N and C terminal regions is located.

Monomers C and D, with broken disulfide bridges, have high thermal stability (high average B factors) with respect to monomers A and B, which each contain a partial disulfide bridge (Table 3). With respect to monomer D, only CYS76 is visible, and we assumed that it did not form because we did not observe the presence of CYS104. The thermal stability of monomer D is the highest, and only the fragments in contact with monomer A are visible.

The B-factor values (Table 3) in association with the ASA showed that monomers A and B are closely related conformations (as confirmed by the RMSD values in Table 4) in two slightly different thermal stability statuses due to the differences in the contact interface of the crystal packing (ASAcrys) of the monomers (143.65 A^2^, considering that an ASA Gly residue is approximately 186 A^2^). In the disulfide bridge, we observed a differential occupancy between A and B monomers with a tendency toward the A (75%) conformation, although the functional meaning of this is unclear. The disulfide bridge proportions on the trimers may induce equilibrium of the conformational forms (with slightly different vibrational motion states) to generate differences during the crystallization process, affecting the crystal lattice array. The absence of a disulfide bridge in monomers C and D (and trimers C and D) induces a notable change in the vibrational motion state properties, observed as high B-factor values. We observed that monomer C has enough ASAcrys, halting the vibrations. Trimers with a high vibrational motion state due to broken disulfide bridges are included on the crystal lattice in spaces where coupling to the surfaces can occur, but with fewer ASAcrys. The vibrational motion state properties of the slightly different conformations (with marginal differences observed in the secondary structure) can induce the formation of a crystal lattice complex such as the P63 space group (Figure S2). In relation to the biological function, the vibrational motion state properties probably condition the protein functions through a clocking mechanism.

Previous studies with the Tv-PSP1 protein demonstrated that the high resistance to denaturation with the urea gradient determined the trimer presence until 7 M urea [17]. The monomers assemble around a three-fold axis, forming a large interaction with each trimer. Two BSA interfaces of the monomer are intended for trimerization, covering 28–30% (1904–2079 Å2) of the ASAm. In other L-PSP1 proteins, the central area of the trimers contains a reduced cavity. These data suggest that the disulfide bridge does not function to maintain the trimer/fold protein, so may be a functional characteristic of Tv-PSP1. The structures show a potential second disulfide bridge between CYS18 and CYS101 from different monomers of the same trimer, which did not formed but are in close contact; this requires further studies to determine. To gain insight to understand the functions of the Tv-PSP1 protein, we performed in silico experiments: first, we aimed to determine the possible contact regions with the mRNAsl proposed by REMSAand intrinsic fluorescence experiments; second, we wanted to obtain information about the functions of the disulfide bridges and their relationship with the vibrational motion state of the thermal stability; third, we wanted to obtain information on the functions as an RNA endonuclease, a mechanism associated with no natural condition. As a result, we observed two conformational states: one with a disulfide bridge (with low B factors) and two without disulfide bridges (high B factors). Apparently, the disulfide bridge induces an increase in vibration motion state not related to the oligomeric stability, but this has an unclear biological function. 

### 3.2. Stem-Loop Structure Interacts with Tv-PSP1

We previously reported a perchloric-acid-soluble protein (PSP) from *T. vaginalis* named Tv-PSP1; this protein has a trimeric structure and may possess a putative ribonuclease function [17], although additional evidence suggests conflicting results due to the purification process with derivatization with chlorine [18,19]. Despite these observations, we do not discard the possible uncommon mechanisms associated with a new process not previously observed, such as functional protein chlorination, even though not necessarily mediated by chorine but potentially with a conformational change induced by other proteins on the regulatory RNA complex. This activity is important because it might affect the key gene expression that may be involved in parasite pathogenesis by recognizing sequences or stem-loop structures such as ERE-like and IRE-like found in the UTRs of the target mRNAs.

**Figure 1:**
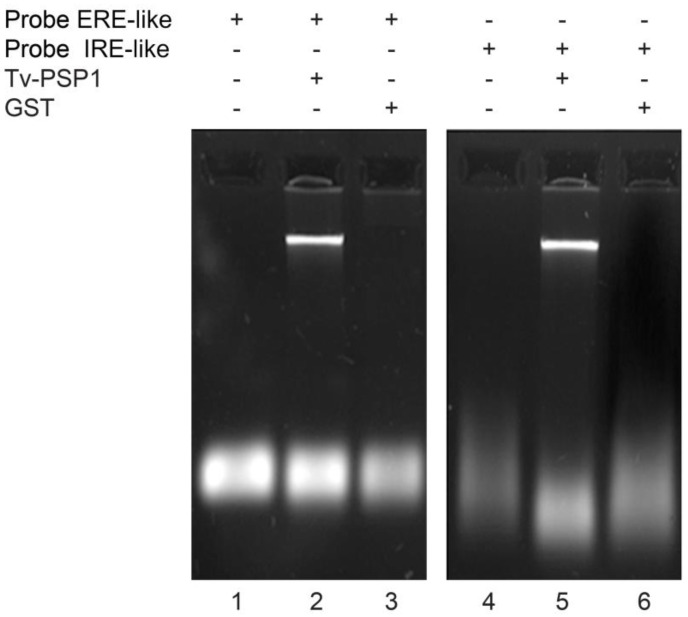
**Interaction between Tv-PSP1 and hairpin-loop mRNA elements.** Line 1, free probe ERE-like. Line 2, free probe ERE-like incubated with Tv-PSP1 trimer. Line 3, free probe ERE-like incubated with GST. Line 4, free probe IRE-like. Line 5, free probe IRE-like incubated with Tv-PSP1 trimer. Line 6, free probe IRE-like incubated with GST.

To determine whether Tv-PSP1 binds to the stem-loop sequences of mRNA, we performed electrophoretic mobility shift assays (REMSA) and determined intrinsic fluorescence. We observed a decrease in mobility due to complex formation between Tv-PSP1 and both stem-loop sequences (Figure 1, Lines 2 and 5). In contrast, we did not observe any complex formation between the stem-loop sequences and an unrelated glutathione-S-transferase (GST) protein (Figure 1, Lines 3 and 6). These results showed that Tv-PSP1 binds stem-loops sequences, suggesting that it may be attached to the RNA molecule. 

**Figure 2:**
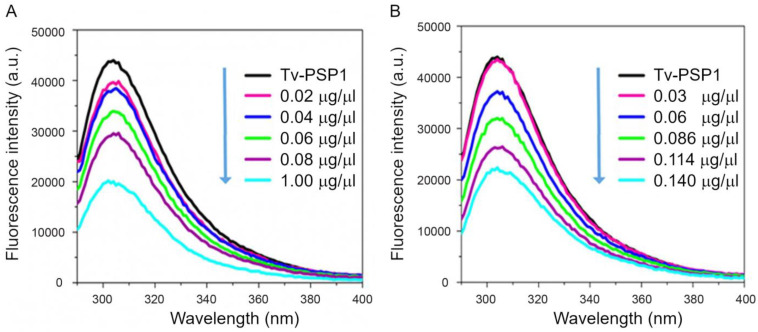
**Fluorescence spectra Tv-PSP1–RNA.** (**A**) Tv-PSP1 fluorescence quenching spectrum with 0.02, 0.04, 0.06, 0.08, and 0.10 µg/µL RNA ERE-like and (**B**) Tv-PSP1 fluorescence quenching spectrum with 0.02, 0.06, 0.086, 0.114, and 0.14 µg/µL RNA IRE-like. All samples were analyzed at 310 K and λ_exc_ = 280 nm.

Proteins exhibit intrinsic fluorescence due to the presence of aromatic amino acids. The Tv-PSP1 protein sequence contains seven tyrosines, which was helpful for our analysis. We obtained the Tv-PSP1 fluorescence intensity from 290 to 400 nm in the absence or after the addition of each stem-loop ERE-like (Figure 2A) or IRE-like (Figure 2B). The intrinsic fluorescence emission spectrum of the Tv-PSP1 protein alone exhibited a maximal emission at 305 nm, which we attributed to Tyr residues. Increasing the ERE-like or IRE-like concentrations from 0.02 to 0.140 µg/mL induced progressive decreases in Tv-PSP1 intrinsic fluorescence intensity (Figure 2A,B, respectively). We attributed this phenomenon to fluorescence quenching by Tv-PSP1 when the complex formed in both systems. 

### 3.3. Tv-PSP1 Trimer Molecular Dynamics and Disulfide Effects on Structure

**Figure 3:**
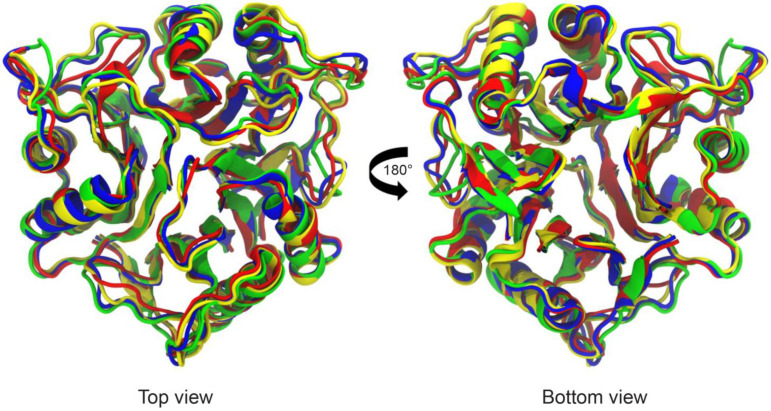
**Molecular dynamics cluster of α-carbon structural superposition.** Tv-PSP1 trimer. **Left** (top surface): Region of convergence of three loops L7. **Right** (down surface): Tv-PSP1 trimer bottom is opposite side of convergence of N and C terminal regions. Proteins are shown as a schematic. Color code: Tv-PSP1 A (blue), Tv-PSP1 B (red), Tv-PSP1 C (yellow), and Tv-PSP1 Cb (green).

We simulated each Tv-PSP1 trimer structure with 100 ns in triplicate using molecular dynamics with charmm36 parameters in Gromacs 2019-2 software. We determined all structures through crystallographic studies: two of them (Tv-PSP1 A and B) have a single disulfide bond (CYS76-CYS104) with different B factors; another two (Tv-PSP1 C and D) have no evidence of any disulfide bond; and the last structure (Tv-PSP1 D) has a partial subunit due to its high vibrational motion state. We simulated an additional Tv-PSP1 trimer structure (Tv-PSP1 Cb), which appears to have two disulfide bonds (one internal within the CYS76-CYS104 folding and one between different chains (CYS18-CYS101). By the end of the triplicate 100 ns molecular simulations, we joined the last 40 ns of each system simulation to a single set of coordinates; we thus obtained a representative structure for each system. All systems studied herein presented small differences among them (the largest difference was 1.31 Å) (Table 5) (Figure 3). 

**Figure 4:**
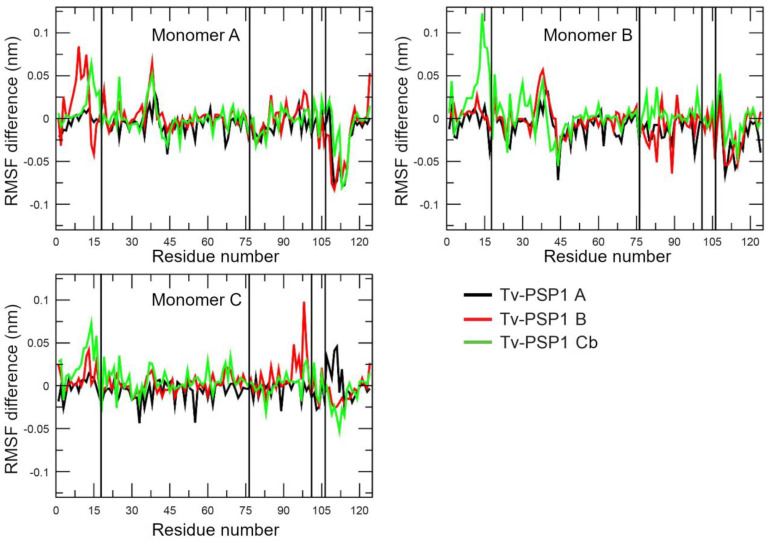
**RMSF differences between all systems against Tv-PSP1 C (no disulfide bonds) by monomer.** Vertical lines show cysteine positions involved in disulfide bonds.

The question remained as to whether the disulfide bonds have any effect on the trimeric Tv-PSP1 structure. To answer this question, we determined the average root mean square fluctuation (RMSF) per residue on the last 40 ns of each simulated system. We averaged each value for each system and compared the results against the system without any disulfide bond (we subtracted each average value for each residue from the system without a disulfide bond). For example, we obtained the RMSF value for residue 1 as: Residue 1 of Tv-PSP1 A minus that of Tv-PSP1 C. A positive value indicated that Residue 1 on Tv-PSP1 A had a larger value than Residue 1 on Tv-PSP1 C, and vice versa. Figure 4 shows the obtained RMSF values.

**Figure 5:**
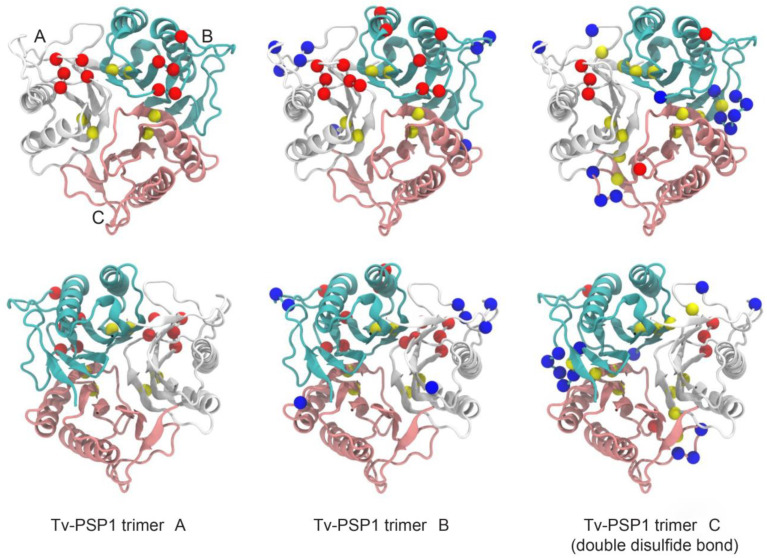
**Residues with RMSF above 0.05 nm (blue spheres) and below −0.05 nm (red spheres) mapped on each representative Tv-PSP1 structure.** Disulfide bonds are shown as yellow spheres. **Upper row** shows structure from top view (top surface), and **lower row** shows bottom view (down surface). Cyan, white, and pink indicate the monomeric subunits of the trimers. Figures were drawn in VMD [39].

As shown in Figure 4, the RMSF fluctuations were different for each structure even when they resembled each other (RMSD values are provided in Table 5). To analyze the RMSF values, we used a threshold of +/− 0.05 nm; hereinafter, our discussion is based on residues within this threshold. As expected, residues with a negative RMSF had a proportion that showed more movement on the Tv-PSP1 C with no disulfide bonds (27 residues) compared with those residues that had a positive RMSF value (19 residues). Table 6 shows the residue numbers on each monomer for each system, and Figure 5 shows the mapping of those residues on the trimeric structure to provide a better visualization.

Tv-PSP1 A did not produce RMSF values greater than those of Tv-PSP1 C on any monomer. Tv-PSP1 B and Cb showed residues (13, 14, 16, and 38) that had more movement compared with Tv-PSP1 C. We noted some common residues that had a larger movement on monomers A and B of Tv-PSP1 C (no disulfide bonds) than those on the other studied systems (110, 111, 113, 114, and 115). The molecular dynamics simulations showed that all residues with positive or negative values in the RMSF comparison are located on loops (L7, L1, L4, L3, and L6), except for two residues on helix 2. The residue loops with RMSF differences repeated between subunits and contained the highest number of residues are the L7 and L1. Loops 3 and 4 and the H2 residue are a few of the residues exposed to the surface or are located on the tip of long loops (as L3), which we considered less relevant (37, 38, 44, 83, and 89) (Table 6). The distributions of residues are shown in Figure 5.

In the Tv-PSP1 crystal structures, L7 is the trimerization loop of the up surface (top side), and L1 is the catalytic loop with the Tyr17 residue (2-iminobutanoate/2-iminopropanoate deaminase activity reported on L-PSP proteins, EC:3.5.99.10). The secondary structure of the L1 is similar to the other reported crystal structure loops, having flexibility and mobility properties, surrounding the possible active site cavity. The L7 in the Tv-PSP1 crystal structures (A, B, and C) show an increased B factor, but the values are lower in the structures with a disulfide bridge, and this loop surrounds the catalytic cavity in the opposite position to L1. Using molecular dynamics simulations, we detected three points with high B factors in the A and B structures (L1, L3, and L7), showing that these movements are present in structure C as well, although this structure has an elevated B factor in general due to the broken disulfide bridge. Additionally observed in structure C was an L7 with an increased B factor all along the loop, as well as other structure points with increased B factors compared with those of the A and B structures. However, in the molecular dynamics simulation, all trimer systems had similar vibrational motion state properties, and we observed the variations on the flexible loop (L1) and the trimerization loop (L7), as is shown in the positive (+) rows in Table 6, with a few relevant residues with positive values (L1 on monomer B) that are higher than the cut off (RMSF values below (0.05). L7 variations in the vibrational motion state properties are possibly associated with the disulfide bridge, located on the previous beta strands five (Cys104) and four (Cys76). When this bridge is broken, the vibrational motion state properties are increased and spread in all structure due to the release of L7. 

With respect to the Tv-PSP1 Cb trimer system with two disulfide bridges, we observed positive values (+) on L1 (an attached system allows major movement to the flexible loops) and negative values on loop L7, but with major restriction compared with that of other systems. These data suggest that L7 may be a special secondary structure with unusual thermal stability properties. However, we observed a general discrepancy or asymmetric trajectory in the molecular dynamics simulation that was hard to explain: the subunit C on all systems had less positive and lower values. The residues that presented changes (above 0.05 or below -0.05 nm RMSF values) were primarily located on the top side of Tv-PSP1. In the next section, we show that this is the opposite region from where we propose that RNA may bind to Tv-PSP1 (Figure 5). 

### 3.4. Tv-PSP1 Trimer-Element Hairpin-Loop (IRE) Interaction: Docking Studies and Electrostatic Calculations

To determine which portion of the Tv-PSP1 structures binds to the IRE RNA hairpin human structures (see Section 2.7), we conducted protein–IRE docking studies. We obtained four Tv-PSP1 structures from clustering analysis in the molecular dynamics simulation (Tv-PSP1 trimer A, trimer B, trimer C, and trimer Cb). We performed docking studies using IRE structures and these mentioned four Tv-PSP1 structures. ERE structures have not yet been reported; therefore, for these docking studies, we only used the IRE structures. This allowed us to determine the possible binding sites of Tv-PSP1 and IRE structures, as well as the energetic contributions to the binding in the Tv-PSP1–IRE complex.

**Figure 6:**
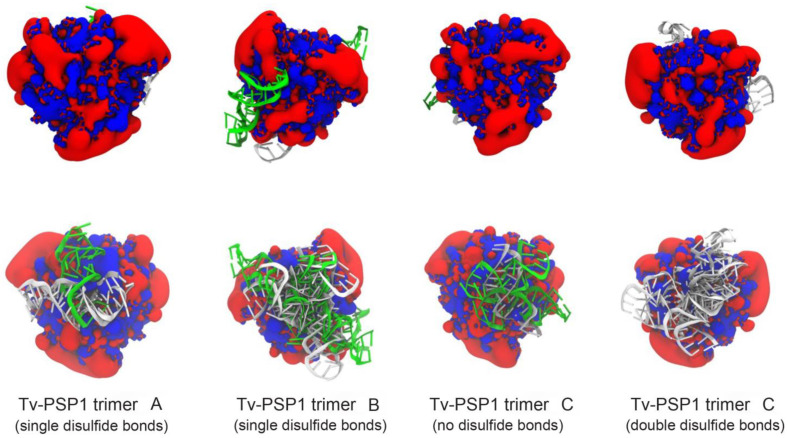
**Tv-PSP1 electrostatic potential (+/- 2 ev) for all studied systems.** RNA molecules with an attractive interaction energy are shown as schematics (1AQO in white, 1NBR in green). **Top row** shows top view (up surface); **bottom row** shows bottom view (down surface). Figures were created with VMD [39].

We performed protein–IRE molecular docking studies to identify and understand the putative Tv-PSP1–IRE binding site. We energy-minimized all complexes, which we used to estimate their binding free energy. We observed that all favorable Tv-PSP1–IRE interactions (negative binding free energy) occurred around the same region of the trimeric structure (Figure 6). The Tv-PSP1 trimer structure electrostatic potential is positive in the RNA interaction region (found in those favorable structures), which may explain the obtained results (Figure 6). 

**Figure 7:**
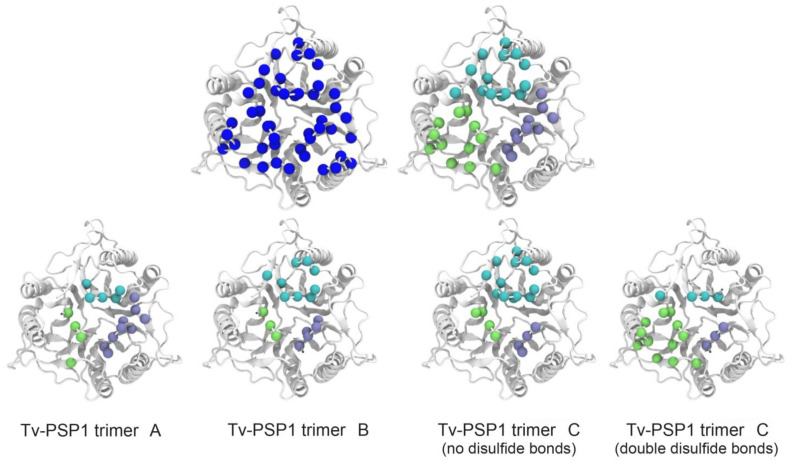
**Consensus positions for the studied systems bound to RNA.** Spheres show residues (α-carbons) that were found to interact with RNA. **Upper row** shows consensus positions (nonredundant positions based on the four systems: **MSKVIS**-X_15_-**LCDR**-X_36_-**KAAG**-X_58_-**YK**) in dark blue and per chain (cyan, chain A; green, chain B; purple, chain C). **Bottom row** shows positions found interacting with RNA for each system. Figures were created with VMD [39].

We determined the IRE binding site by selecting the amino acids that were located at 5 Å or less from the IRE on every studied complex. Sixteen amino acids remained constant between the Tv-PSP1 and IRE structures. These amino acids might correspond to the binding motif between Tv-PSP1 and IRE structures. These mapped interactions of amino acids at the Tv-PSP1 trimer surface were formed by residues **MXKXI** (1 to 5), **LCDRT** (22 to 26), **AAGY** (62 to 65), and **YK** (123 and 124) (Figure 7). Aspartic acid, located on position 24, may play a significant role in RNA recognition of Tv-PSP1. Possible mutations on position 24 for residues with a positive or neutral charge may improve Tv-PSP1–RNA binding.

Electrostatic interactions are an important part of the recognition and binding between molecules. Molecular interactions are attractive or repulsive forces between molecules, which are mainly involved in the regulatory processes of RNA to control gene expression^30^. To determine whether the Tv-PSP1 trimer–IRE interaction is favorable, we analyzed the energy values to evaluate the electrostatic contributions. We determined ΔG_elec_ and ΔG_non-elec_ from the binding free energy of these complexes. The energy values of the studied Tv-PSP1 trimer–IRE (1AQO) are shown in Table 7, where trimers A and B are similar oligomers, highlighted by their different B factors. Trimer C has no disulfide bonds (and higher B factors), and trimer Cb has two bonds. This condition was not experimentally observed, but possibly forms under unclear conditions in the parasite cell. 

All complexes present favorable Coulombic interactions, whereas nonelectrostatic interactions are less favorable. However, solvation energy is positive, indicating that desolvation of the individual molecules is unfavorable because electrostatic interactions have a desolvation penalty. For the Tv-PSP1 trimer–IRE (1NBR) complex, the energy values are shown in Table 8 under same conditions. We also observed that the interaction process is motivated by electrostatic contributions. In contrast, certain IRE poses occur on the top and side of the Tv-PSP1, which exhibit positive energy values.

## 4. Discussion

Eukaryotic cells possess several proteins capable of binding RNA to form complex networks of RNA machineries that participle in the cellular regulation of several RNA-processing events such as transcription, translation, splicing, and epigenetic control. These processes are also essential for regulating gene expression [2]. Nucleic acids function through interactions with proteins; therefore, we need to understand the recognition mechanisms that occur in protein–RNA interactions. Unlike DNA, RNA shows a wider variety of conformations and forms, which determine the interaction between RNA and proteins. The use of diverse experimental techniques such as X-ray crystallography, intrinsic fluorescence, and REMSA in conjunction with in silico techniques, such as molecular dynamics and protein–RNA docking simulations, allows us to predict the putative binding regions of RNA to protein.

Identifying RNA-interacting proteins that may recognize molecules of single or double-strand RNA is an important step in understanding these putative mechanisms. In *T. vaginalis*, cytoplasmic IRP-like proteins may interact with IRE-like stem-loop structures located at the UTRs of certain mRNAs, such as *tvcp4* and *tvcp12* from cysteine proteinases as well as Tv-eIF-5A, which are capable of binding to specific RNA sequences at the UTRs of *tvcp39* in ERE-like stem-loop structures [6,8,9].

The mammal L-PSP homologue protein (UK114/HRSP12) recognizes RNA sites and participates in RNAm regulation, mediating the interaction of protein complexes [20].

The RNA degradation mechanisms in the parasite have not yet been described. In other parasites, such as *Entamoeba histolytica*, a member of the PSP family (EhL-PSP), two protein complexes are associated with RNA degradation: the cytoplasmic P-bodies (catalyzed by deadenylases) and exosome vesicles (catalyzed by endo-exoribonuclease) [16]. Therefore, *T. vaginalis* Tv-PSP1 protein highly likely maintains a conserved structure function associated with RNAm regulation working in not-well-identified RNAm protein complexes until now (potentially the evolved homologues complexes).

However, the main objective of this study was to determinate whether the Tv-PSP1 protein is capable of recognizing and binding RNA stem-loop structures, which have already been described in the UTR end of the parasite’s messenger RNAs. These stem-loop structures are important in gene regulation, controlling the cytotoxicity of the parasite, which include the *tvcp39*, *tvcp4*, and *tvcp12* genes [9,10].

L-PSP structure proteins showed putative active sites, which focus on small ligands, such as free fatty acid [44], benzoate molecule [45], or ketobutirate [46]. These molecules bind to the functional catalytic cavities located between monomers of the trimeric structure. However, to the best of our knowledge, no study describes their possible interaction with larger molecules such as the stem-loop structure of RNA. However, the interaction of mammalian L-PSP with the aRNA signature was reported, but the aRNAm structure was not mentioned [20].

Volz et al. reported that crystals of the YjgF protein from *Escherichia coli* have three putative active sites located between the three interfaces of the trimer consisting of four invariant amino acids: Gly31, Asn88, Arg105, and Glu120, which are also conserved in the Tv-PSP1 protein [17,47]. However, they are not related to the recognition of or the interaction with RNA, as we determined in our molecular docking study. The Tv-PSP1 protein likely has some other functions that cannot be ruled out, because the family to which YER057c/YjgF/Uk114 belongs includes proteins whose functions have not been established. They are considered multifunctional, as reported in different organisms. Furthermore, they have different biological functions [11,12,13], and many of them are related to RNA mechanisms [14]. We determined that the Tv-PSP1 trimer structure, where the interaction with RNA is positively charged, agrees with previous findings for a protein from *E. coli* with a positively charged, trimeric conformation of the ST0811 surface, thus suggesting that this may favor RNA molecule interactions [47]. Electrostatic interactions can facilitate the recognition of their active site for the binding of molecules.

We conducted computational calculations to determine the energy of the electrostatic interactions in the complexes through molecular dynamics simulations to determine whether electrostatic interactions play an important role in the recognition of molecules. Electrostatic interactions play a role in the initial attraction between RNA and positively charged residues [48]. The presence of positively charged amino acids (Lys3, Arg25, Lys62, and Lys124 on each Tv-PSP1 monomer) on the reported binding site is expected to be higher because they may interact with the negatively charged RNA phosphate groups [49]. Additionally, Tyr94 and Tyr123, both on the bottom interface surface region as neighbors of the mentioned positively charged amino acids, and Try17, on the possible active site cavity, may be the main residues responsible for the quenching observed on the fluorescence, and Phe84 located on the possible active site cavity neighbor of the Tyr17 and Arg102 may contribute.

The recognition of the appropriate binding sites is facilitated by the presence of electrostatic interactions; charged amino acids considerably contribute to RNA binding even at distances of up to 11 Å [48]. Regularly, the dominant amino acid in the RNA–protein interaction is arginine, which is most frequently involved in all interaction modes (electrostatic, hydrogen bonding, stacking, van der Waals, and hydrophobic). Another frequently involved amino acid is lysine. Both are amino acids that exhibit a higher propensity of binding with nucleic acids [49]. For example, electrostatic interactions based on the complex of the protein U1A and U1 hairpin II RNA are positively charged amino acids (Lys55 and Lys95), which interact with the phosphate backbone of the RNA loop and stem.

These examples show that understanding the role of electrostatic interactions is important to better understand the mechanism of RNA–protein interactions. The electrostatic interactions of well-positioned, positively charged residues are important for both the initial formation and maintenance of the complex [48]. The presence of positively charged amino acids on the Tv-PSP1-RNA interface is notable: **MSKVIS**-X_15_-**LCDR**-X_36_-**KAAG**-X_57_-**YK** (Figure 7). However, much remains to be investigated in terms of the role of these interactions in the formation of RNA-Tv-PSP1 complexes.

Studies of protein–nucleic acid interactions study are simplified by all perceptible fluorescence arising from the protein. Changes in the fluorescence emission spectrum of a protein upon binding to RNA can often be used to determine binding. Thus, change in fluorescence is assumed to be the effect of binding to a nucleic acid [46]. This is consistent with the decrease in the fluorescence intensity of Tv-PSP1, which we observed with an increase in ERE-like or IRE-like concentration. This result is also consistent with the results of complex formation from the electrophoretic mobility shift assay.

## 5. Conclusions

The structure of Tv-PSP1 shows unusual thermal stability of the quaternary structure, suggesting the presence of at least two kinds of trimers with two conformations in solution: one with a disulfide bridge (CYS76-CYS104) and the other without (elevated thermal stability). This thermal stability is potentially associated with the function of this protein, but it is not yet clear how it works. We propose two main possibilities: (i) a function related with protein exchange (with high thermal stability being a signal) associated with the RNA-processing regulation; (ii) the status of the thermal stability determines the functions of a hypothetical multifunctional Tv-PSP1, separating the catalytic functions (lower thermal stability) from the gene regulation functions (elevated thermal stability). Elevated thermal stability is convenient for binding mRNA and allowing the docking of the stem-loop RNA structures. The conformation with disulfide bridge is capable of binding RNA as well, but this may be more restricted and allow other proteins act in RNA regulation.

The Tv-PSP1 protein from *T. vaginalis* is capable of binding stem-loop structures from RNAm. For the first time, we suggest that an interaction may occur between RNA and Tv-PSP1, as indicated by the results of our experimental and computational studies. The changes in the fluorescence spectrum of Tv-PSP1 upon binding to RNA indicated this interaction. We determined the binding modes between Tv-PSP1 and the stem-loop structure through computational studies such as the electrostatic determinations, which is important for understanding the molecular recognition of Tv-PSP1 in *T. vaginalis*. As shown in all analyzed complexes, a favorable Coulombic interaction indicated recognition and binding between molecules. The results of molecular docking studies with Tv-PSP1–IRE showed favorable interactions that occurred around the same region of the trimeric structure.

This is the first study to propose a site of interaction between a Tv-PSP1 protein and RNA structures. Additionally, we are currently working on obtaining the X-ray crystal structure of this protein–RNA complex to determine the conformation of the binding site between the protein and RNA, which is an important step in our understanding of the gene regulation mechanisms in *T. vaginalis*.

## Figures and Tables

**Table 1:** Data collection, phasing, and refinement statistics.

PDB ID	7KGC
**Data collection**	
Wavelength (Å)	0.97
Space group	P6_3_
Cell dimensions	
a, b, c (Å)	81.9, 81.9, 129.3
α, β, γ (°)	90.0, 90.0, 120.0
Resolution (Å)	25.0–1.95 (2.0–1.95)
Unique reflections	35 775 (2529)
I/σI	14.1 (2.4)
R_merge_ (%)	8.2 (44.6)
CC_1/2_	0.99 (0.80)
Completeness (%)	99.7 (99.9)
Multiplicity	3.5 (3.5)
Mosaicity (°)	0.3
**Refinement**	
Resolution (Å)	25.0–1.95
R (%)	19.5
R_free_ (%)	24.1
Number of atoms	
Protein	3151
TRIS	24
Water	295
B factors (Å^2^)	
Protein	37.7
TRIS	50.1
Water	37.9
All atoms	37.0
Wilson plot	23.7
RMSD	
Bond lengths (Å)	0.017
Bond angles (°)	1.474
Ramachandran plot	
Most favored regions (%)	97.78
Additional allowed regions (%)	1.97
Disallowed regions (%)	0.25

Statistics for highest-resolution shell are shown in parentheses.

**Table 2:** Tv-PSP1 trimer assembles using molecular dynamics simulations.

Protein	Disulfide Bond
Tv-PSP1 trimer A	CYS 76-CYS 104 (Chain A) CYS 76-CYS 104 (Chain B) CYS 76-CYS 104 (Chain C)
Tv-PSP1 trimer B	CYS 76-CYS 104 (Chain A) CYS 76-CYS 104 (Chain B) CYS 76-CYS 104 (Chain C)
Tv-PSP1 trimer C	Without disulfide bridge
Tv-PSP1 trimer Cb	CYS 76-CYS 104 (Chain A) CYS 76-CYS 104 (Chain B) CYS 76-CYS 104 (Chain C) CYS 18 (Chain A)-CYS 101 (Chain B) CYS 18 (Chain C)-CYS 101 (Chain A) CYS 18 (Chain B)-CYS 101 (Chain C)

**Table 3:** B-factor values and PISA analyses of Tv-PSP1.

B Factor Value Average by Residue of Main Chain Atoms				ASA of Interfaces and Surfaces on the Crystal **			
	AMC	Minimum	Maximum	Interfaces	A	B	C
**A** *	19.70	12.60	49.74	ASAbio	1904.80	2079.10	2015.7
**B** *	24.05	14.67	52.98	ASAcrys	1275.91	1132.26	788.26
**C**	55.85	30.73	88.48	**Surface**	**A**	**B**	**C**
**D**	73.41	38.99	102.85	ASAm	6756.50	6910.50	6790.90
				ASAtrimer	14,555.4	14,494.2	14,325.5

ASAbio: total contact interfaces BSA between subunits of same trimers; ASAcrys: contact interface BSA of crystal packing by monomer; ASAm: ASA surface of each type of monomer; ASAtrimer: ASA surface from each trimer; * monomeric subunits with disulfide bridge; ** monomer D was not considered; few contacts were observed, exclusively with monomer A.

**Table 4:** General comparison of data of Tv-PSP1 crystal monomers and trimers.

RMSD between Monomers				RMSD between Trimers		
		B	C		TB	TC
A	RMSD (atoms)	0.227 (105)	0.310 (107)	TA	0.241 (315)	0.361 (318)
A	RMSD (all atoms)	0.750 (124)	0.683 (124)	TA	0.760 (372)	0.676 (372)
B	RMSD (atoms)	0.00	0.352 (93)	TB	0.00	0.402 (267)
B	Atoms (all atoms)	0.00	0.951 (124)	TB	0.00	0.980 (372)
Cut off 0.6 Å.				Cut off 0.6 Å.		

RMSDs between monomers and trimers are shown at two values: one with the number of CA atoms adjusted by the cut off (atoms) and other considering all CA atoms (all atoms).

**Table 5:** α-Carbon RMSD of the molecular dynamic cluster center structure of each Tv-PSP1 trimer.

	Tv-PSP1 A	Tv-PSP1 B	Tv-PSP1 C	Tv-PSP1 Cb
**Tv-PSP1 A**	0.0	1.08	0.97	1.13
**Tv-PSP1 B**		0.00	1.22	1.31
**Tv-PSP1 C**			0.00	1.19
**Tv-PSP1 Cb**				0.00

**Table 6:** Secondary structures with outstanding RMSF values for molecular dynamics cluster.

	A	B	C	
**Tv-PSP1 A**	No	No	No	RMSF (+)
**Tv-PSP1 A**	L7	L7	No	RMSF (−)
**Tv-PSP1 B**	L1	No	No	RMSF (+)
**Tv-PSP1 B**	L7	L7	No	RMSF (−)
**Tv-PSP1 C**	Ns	L1	L1	RMSF (+)
**Tv-PSP1 Cb**	L7	Ns	Ns	RMSF (−)
	+		−	Differences

L1 = correspond to residues 9, 11, 12, 13, 14, 15, 16, 17, 18, and 19. L7 = correspond to residues 109, 110, 111, 113, 114, and 115. No = not observed. Residues with positive and negative RMSF values below 0.05 and above −0.05 based on Figure 4. Negative values (−) indicate that residues had major movement on trimer system Tv-PSP1 C (without disulfide bonds); positive values (+) indicate residues with less movement on trimer system Tv-PSP1 C (or major movement on other systems A, B, and Cb). Ns = not significant.

**Table 7:** Binding energy (ΔG_b_) summary of most favorable Tv-PSP1 trimer structure obtained from molecular dynamics simulations with IRE from structure 1AQO, determined by APBS and VMD.

Complexes	ΔG_solv_ (kJ/mol)	ΔG_coul_ (kJ/mol)	ΔG_non-elec_ (kJ/mol)	ΔG_b_ * (kJ/mol)
**Tv-PSP1 trimer A IRE**	142	−849	−37	−744
**Tv-PSP1 trimer B IRE**	178 185 212 214 189 129	−823 −698 −726 −686 −614 −252	−37 −35 −33 −35 −34 −26	−682 −548 −547 −507 −459 −149
**Tv-PSP1 trimer C IRE**	215 198	−664 −639	−34 −35	−483 −477
**Tv-PSP1 trimer Cb IRE**	233 218 273 340 320	−788 −479 −479 −409 −381	−44 −31 −40 −43 −39	−599 −292 −246 −112 −100

* ΔG_b_ = ΔG_solv_ + ΔG_coul_ + ΔG_non-elec_.

**Table 8:** Binding energy (ΔG_b_) summary of most favorable Tv-PSP1 trimer structures obtained from molecular dynamics simulations with IRE from structure 1NBR, determined by APBS and VMD.

Complex	ΔG_solv_ (kJ/mol)	ΔG_coul_ (kJ/mol)	ΔG_non-elec_ (kJ/mol)	ΔG_b_ * (kJ/mol)
**Tv-PSP1 trimer A–IRE**	136 269	−832 −631	−31 −37	−727 −399
**Tv-PSP1 trimer B–IRE**	154 201 188 152	−836 −639 −482 −343	−41 −35 −32 −28	−723 −473 −326 −219
**Tv-PSP1 trimer C–IRE**	296	−46	−37	−213
**Tv-PSP1 trimer Cb–IRE**	177	12	−32	157

* ΔG_b_ = ΔG_solv_ + Δ_Gcoul_ + ΔG_non-elec_.

## Data Availability

https://doi.org/10.5281/zenodo.7429982; https://www.rcsb.org/structure/7KGC.

## Supplementary Materials

The following supporting information can be downloaded at: https://www.mdpi.com/article/10.3390/pathogens12010079/s1, Figure S1: Tv-PSP1 crystal packing; Figure S2: Tv-PSP1 secondary structure and general topology.
