# Supplementary material for: A Putative New Role of Tv-PSP1 Recognizes IRE and ERE Hairpin Structures from Trichomonas vaginalis
Source: Pathogens. 2023 Jan 3;12(1):79. doi: 10.3390/pathogens12010079 (PMC9863245; doi:10.3390/pathogens12010079)
Supplement: Supplementary file 1 [file pathogens-12-00079-s001.zip › S1-S2-FigCaptionSupplementary.docx]

Figure S1. Tv-PSP1 crystal packing. Crystal packing of the hexagonal space group P63 with cell dimensions
a=81.9 Å, b=81.9 Å, c=129.3 Å, and γ=120°. A) Trimer A in Grey surface is around the threefold axis symbol. B)
Trimer D in blue steel color, this trimer is under Trimer A on the same threefold axis. The trimer D on the final
structure is not visible in a large part of the structure, only are visible the fragments in contact with monomer A,
here was built a complete Trimer from previous refinement process to illustrate the position on the crystal.
C)Trimer B in green color is around the threefold axis symbol in the symmetric object of the twofold screw axis
of the cell. D)Trimer C in orange color is around the sixfold axis symbol. Figure was made in VMD program [39]

Figure S2. Tv-PSP1 secondary structure and general topology. A) Tv-PSP1 secondary structure of the
asymmetric unit monomers obtained with VMD program [39]. Marginal differences are observed on the L1 and
L7. B) General topology of the monomer A structure. Beta strands in yellow color, 3-10 helixes in blue color, alfa
helixes in magenta color, turns and coil in green color.
