# Supplementary figures and images for: A Putative New Role of Tv-PSP1 Recognizes IRE and ERE Hairpin Structures from Trichomonas vaginalis
Source: Pathogens. 2023 Jan 3;12(1):79. doi: 10.3390/pathogens12010079 (PMC9863245; doi:10.3390/pathogens12010079)

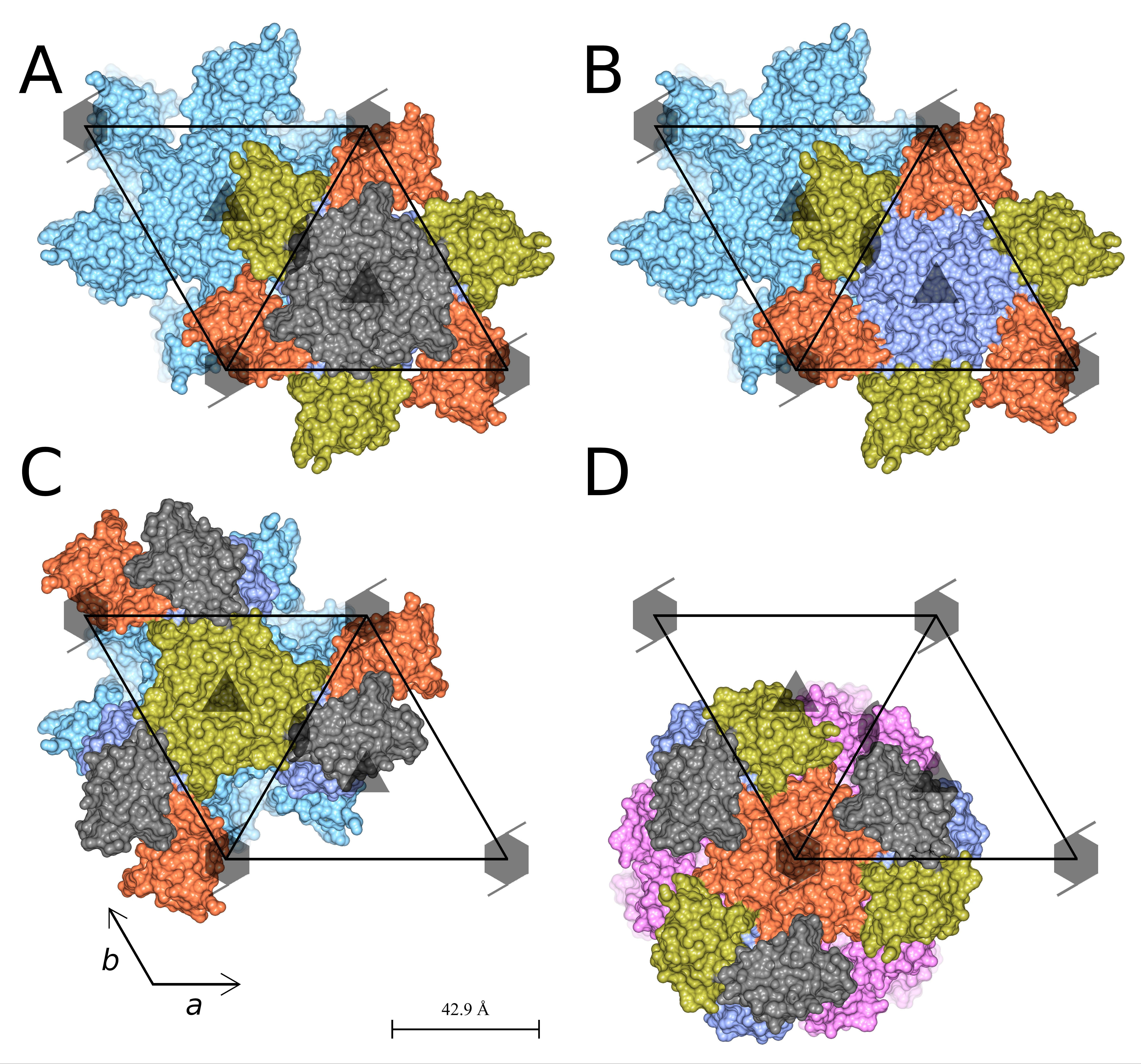

Supplement: Supplementary file 1 [file pathogens-12-00079-s001.zip › FigureS1.jpg]

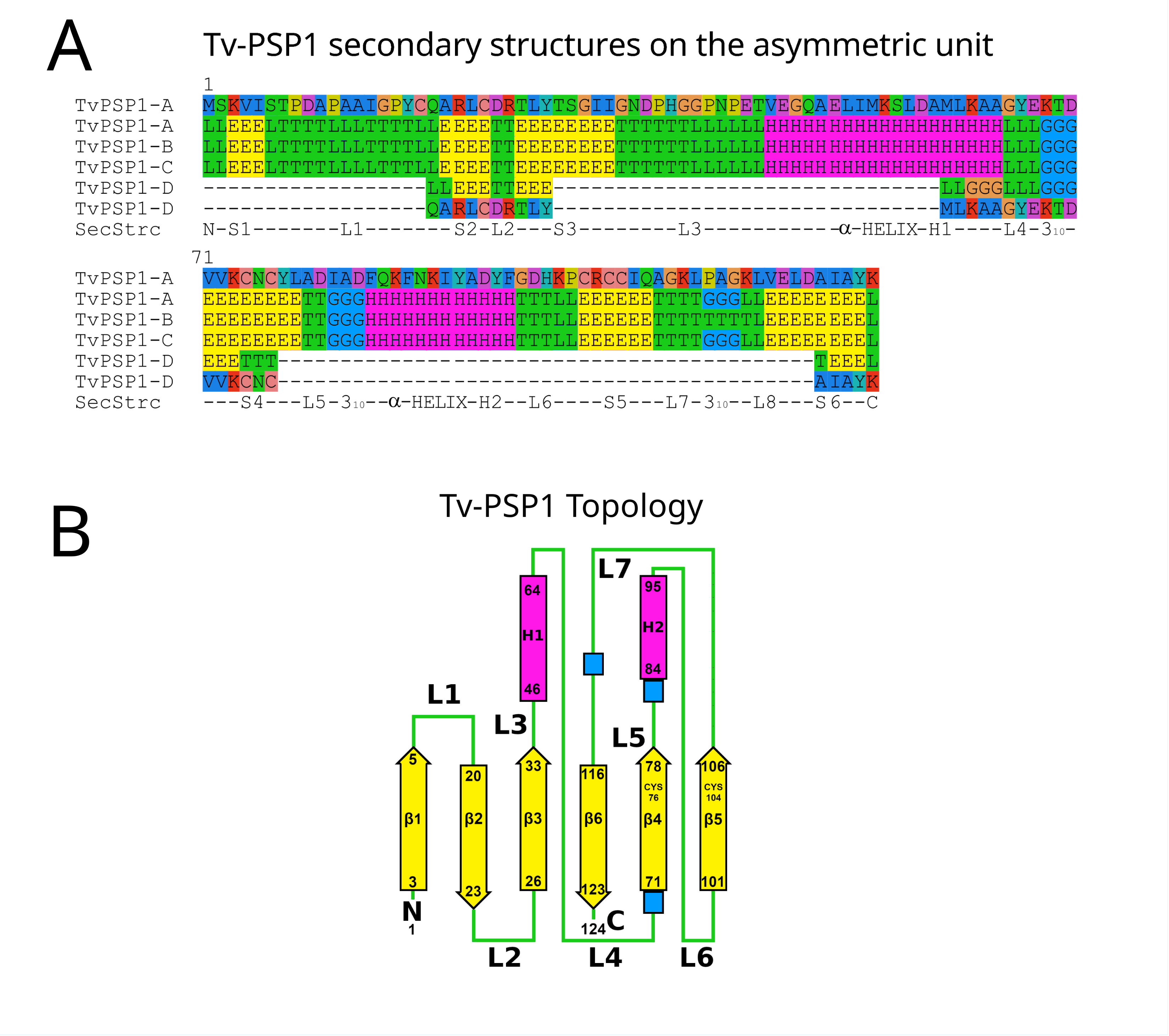

Supplement: Supplementary file 1 [file pathogens-12-00079-s001.zip › FigureS2.tif]
